# Vitamin D and allergic diseases

**DOI:** 10.3389/fimmu.2024.1420883

**Published:** 2024-07-04

**Authors:** Panyu Zhang, Qingxiu Xu, Rongfei Zhu

**Affiliations:** ^1^ Department of Allergy, Tongji Hospital, Tongji Medical College, Huazhong University of Science and Technology, Wuhan, China; ^2^ Institute of Allergy and Clinical Immunology, Tongji Hospital, Tongji Medical College, Huazhong University of Science and Technology, Wuhan, China

**Keywords:** vitamin D, allergic rhinitis, asthma, atopic dermatitis, food allergy, allergen immunotherapy

## Abstract

In recent years, the relationship between vitamin D and allergic diseases has received widespread attention. As a fat-soluble vitamin, vitamin D plays a crucial role in regulating the immune system and may influence the onset and progression of diseases such as atopic dermatitis, allergic rhinitis, and asthma. To understand the underlying mechanisms, we have summarized the current research on the association between vitamin D and allergic diseases. We also discuss the impact of vitamin D on the immune system and its role in the course of allergic diseases, particularly focusing on how vitamin D supplementation affects the treatment outcomes of these conditions. We aim to provide a theoretical basis and practical guidance for optimizing the management and treatment of allergic diseases by modulating vitamin D levels.

## 1 Introduction

Allergic diseases are a result of the immune system’s overreacted response to allergens, with a diverse set of immune cells (such as lymphocytes, mast cells/basophils) and immune molecules like IgE playing a role in the pathogenetic process. Studies suggest that a complex interplay between genetic, environmental, and nutritional factors can lead to the onset of allergic diseases (1). Over the past decades, there has been a dramatic increase in the prevalence of allergic diseases, such as atopic dermatitis (AD), allergic rhinitis (AR), and allergic asthma (AA), posing a significant societal burden (2).

Vitamin D, a fat-soluble vitamin, exists in two forms: D2 (ergocalciferol) and D3 (cholecalciferol) (3). Initially, vitamin D is hydroxylated by the 25-hydroxylase enzyme in the liver to form 25-hydroxyvitamin D (25(OH)D), which is then metabolized in the kidneys by the 1α-hydroxylase enzyme into the biologically active form, 1,25-dihydroxyvitamin D (1,25(OH)2D) (4). Vitamin D primarily acts through the vitamin D receptor (VDR) to regulate calcium and phosphorus balance and maintain bone health (5). Given that mast cells, monocytes, macrophages, T cells, B cells, and dendritic cells (DCs) express nuclear receptors (nVDR) and membrane receptors (mVDR) (6), vitamin D also plays a vital role in modulating immune responses (7, 8).

Recent research has indicated that vitamin D, through its regulatory effect on the immune system, could be involved in the onset and progression of allergic diseases. This article provides a review of the influence of vitamin D on the immune system, the relationship between vitamin D and allergic diseases, and the impact of vitamin D supplementation on allergic outcomes.

## 2 The impact of vitamin D on the immune system

Vitamin D primarily exerts its immunoregulatory effects through the VDR. The expression of VDR in immune cells such as DCs, macrophages, monocytes, and lymphocytes provides a foundation for its role in immune regulation (9, 10).

### 2.1 Vitamin D and innate immune cells

Innate immunity is the body’s frontline defense, swiftly fending off pathogen invasions. Vitamin D showcases distinct impacts on various innate immune cells through different routes.

Vitamin D mainly exerts immunosuppressive effects on human innate lymphoid cells (ILCs), inhibiting the ability of vitamin A-induced ILC2 cells to produce cytokines such as IL-5 and IL-13, and the expression of gut-directed integrin α4β7 induced by vitamin A (11). Vitamin D inhibits the response of ILC3 cells to IL-23 through its receptor, thereby inhibiting the production of cytokines such as IL-22, IL-17F, and granulocyte-macrophage colony-stimulating factor (GM-CSF), while enhancing the expression of genes associated with the IL-1β signaling pathway, converting the production of ILC3 cell factors to the production of innate cytokines, such as IL-6, IL-8, macrophage inflammatory proteins 1α/β (MIFs) (12).

Vitamin D mainly exerts inhibitory effects on eosinophils. 1,25(OH)2D can upregulate the expression of C-X-C motif chemokine receptor 4 (CXCR4) on them, promoting the transfer of eosinophils from allergic inflammation sites to non-inflammatory tissues outside the blood vessels induced by the latter (13, 14), and can inhibit the production of eosinophil mediators, such as major basic protein (MBP), eosinophil peroxidase (EPX), eosinophil cationic protein (ECP), and eosinophil-derived neurotoxin (EDN) (15). In a mouse asthma model, vitamin D reduced the infiltration of eosinophils in the lungs (16).

In mast cells, 1,25(OH)2D can increase the number of VDRs in mast cells, maintain the stability of mast cells, and inhibit the production of inflammatory and vasodilatory mediators mediated by IgE in human mast cells (17, 18). *In vitro* studies have shown that vitamin D upregulates the expression of IL-10 mRNA in mouse mast cells and induces the secretion of IL-10 (19).

Vitamin D enhances the formation of neutrophil extracellular traps (NETs), upregulates the production of IL-4, and downregulates the expression of pro-inflammatory cytokines IL-1β, IL-6, IL-8, and IL-12 in neutrophils (20–22). Some studies have shown that vitamin D induces apoptosis of peripheral blood neutrophils in patients with acute exacerbation of chronic obstructive pulmonary disease (AECOPD) through the p38 MAPK signaling pathway (23).

For NK cells, vitamin D can promote their secretion of IFN-α, making them more successful in exerting antibody-dependent cellular cytotoxicity (ADCC) effects (24).

Monocytes/macrophages can recognize components of bacteria, viruses, and fungi through their surface-expressed toll-like receptors. Vitamin D can form a 1,25/VDR/RXR complex with VDR and retinoid X receptor (RXR) on monocytes/macrophages, promoting the expression of toll-like receptors (25), enhancing the chemotaxis and phagocytosis capabilities of monocytes/macrophages, and inducing the production of antimicrobial peptides (26). Additionally, 1,25(OH)2D can promote the development of macrophages, which play a key role in the phagocytosis and clearance of bacteria, as evidenced by the increased expression of complement receptor immunoglobulin (CRIg) mRNA, protein, and cell surface expression. The phagocytic ability of macrophages treated with 1,25(OH)2D is also significantly enhanced (26, 27). In general, macrophages polarize into different phenotypes under various inflammatory conditions (28). Resting macrophages (M0) become polarized into pro-inflammatory M1-like macrophages (M1) when exposed to stimuli such as lipopolysaccharide (LPS), interferon-alpha (IFN-α), IL-12, and IL-23. These M1 macrophages primarily produce pro-inflammatory cytokines such as TNF-α, IL-23, IL-12, and IL-1β, thereby promoting inflammatory responses. Conversely, IL-4 and IL-10 enhance the development of anti-inflammatory M2-like macrophages (M2), which produce anti-inflammatory cytokines IL-10 and TGF-β, promoting wound healing and maintaining tissue homeostasis (29, 30).

Studies have shown that vitamin D, through the VDR pathway, downregulates the expression of IL-12, TNF-α, and IL-1β in M1 macrophages, as well as the expression of co-stimulatory molecules CD80 and CD86 on macrophages, thereby reducing the macrophages’ ability to stimulate T cells. Simultaneously, vitamin D upregulates the production of IL-10 and TGF-β in M2 macrophages, promoting the differentiation of macrophages towards the M2 phenotype (31, 32). This polarization alleviates the development of allergic diseases such as AR and AD (33–35).

For dendritic cells (DCs), it has been found that 1,25(OH)2D can inhibit the expression of MHC class II and co-stimulatory molecules CD40, CD80, and CD86 on the surface of DCs, thereby reducing their antigen-presenting and T cell-activating capabilities. It also inhibits the release of pro-inflammatory cytokines such as tumor necrosis factor alpha (TNF-α), interferon-gamma (IFN-γ), and interleukin-2 (IL-12) (which influences Th cell differentiation into Th1 cells), and IL-23 (which influences Th cell differentiation into Th17 cells). Additionally, it upregulates IL-10 (an anti-inflammatory cytokine that inhibits Th2-type immune responses) and IL-6, reduces the production of C-C chemokine ligand 17 (CCL17), and inhibits the differentiation, maturation, and chemotactic abilities of DCs (36–41). Moreover, vitamin D promotes the induction of FOXP3 transcription by DCs to enhance the generation of Tregs, thus boosting immune tolerance and reducing allergic reactions (42, 43). Brulefert et al. collected human skin samples to investigate the effects of vitamin D on DCs. They found that vitamin D-induced CD14+ skin DCs significantly increased the production of IL-4 and IL-13, promoting T helper cell 2 (Th2) responses even in the absence of TSLP (44). This seems contradictory, suggesting that the mechanisms by which vitamin D affects DCs need further investigation.

**Figure 1:**
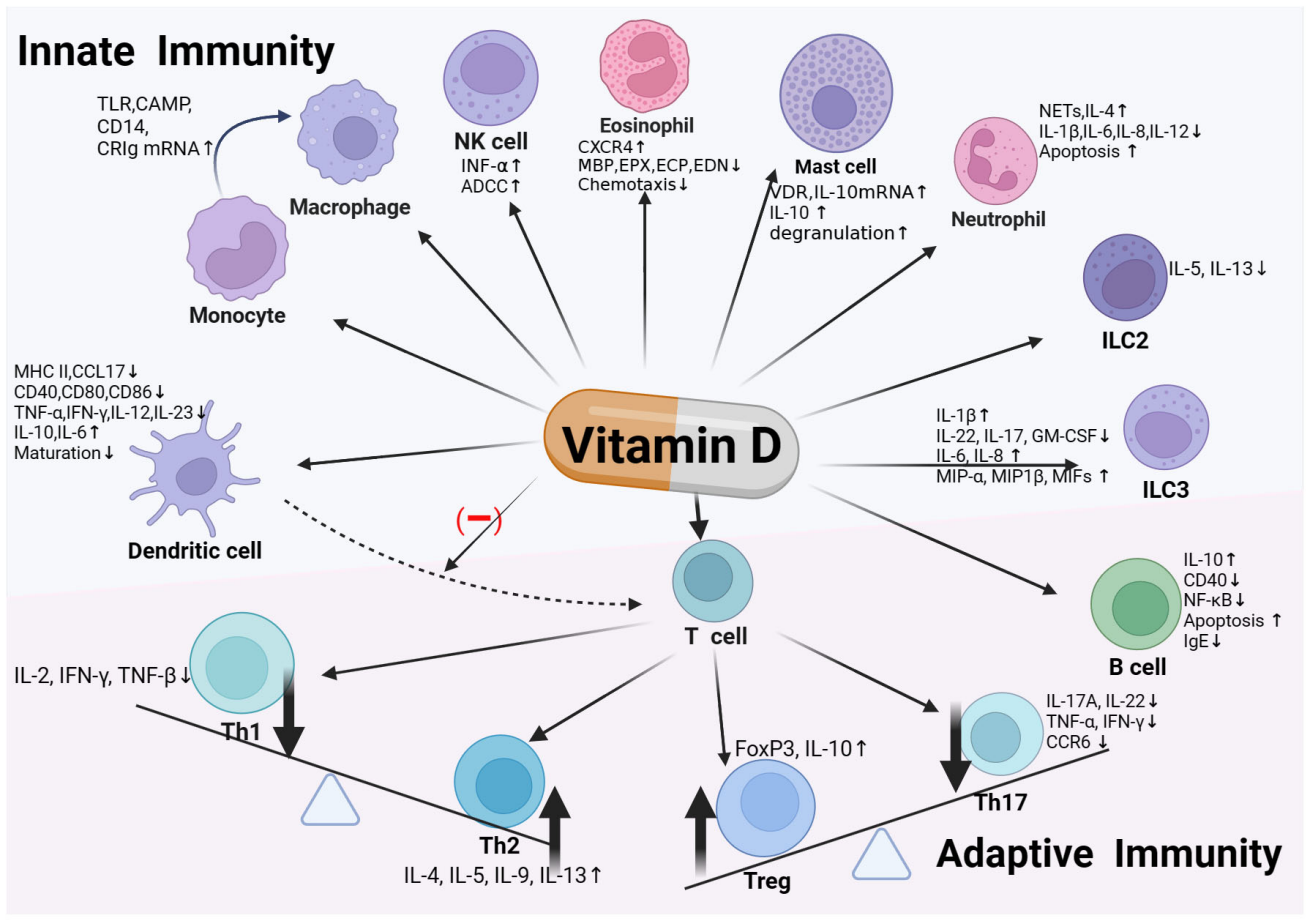
Vitamin D and immune system. Vitamin D exerts immunoregulatory effects by binding to the Vitamin D Receptor (VDR) expressed on various immune cells, including monocytes/macrophages, dendritic cells, innate lymphoid cells (ILC), as well as T and B cells within the adaptive immune system. In innate immunity, vitamin D enhances monocyte/macrophage chemotaxis and phagocytosis, and induces antimicrobial peptide production. It modulates dendritic cell maturation, activation, and chemotactic and immunostimulatory capabilities, along with affecting the functions of ILCs and eosinophils. In the realm of adaptive immunity, vitamin D promotes the development of Th2 and regulatory T cells (Treg), while inhibiting the differentiation and activation of Th1 and Th17 cells. It also modulates B cell activity and IgE production. Through these pathways, vitamin D contributes to maintaining immune homeostasis and preventing excessive inflammatory responses, thereby playing a vital role in preserving human health.

Through the above various ways, vitamin D regulates the function of innate immune cells, playing a crucial role in the body’s first line of defense (**Figure 1**).

### 2.2 Vitamin D and adaptive immune cells

The influence of vitamin D on T cells varies across different subsets, primarily showcasing inhibitory effects on T helper cell 1 (Th1) and T helper cell 17 (Th17) subsets and stimulatory effects on Th2 and regulatory T cell (Treg) subsets. Sloka et al. used an experimental autoimmune encephalomyelitis (EAE) model and *in vitro* cultures of human and mouse cells to demonstrate that 1,25(OH)2D upregulates GATA-3 through a STAT6-dependent mechanism, promoting Th2 cell polarization and inhibits the differentiation of Th1 and Th17 cells and the production of inflammatory cytokines (45). Zhang et al. constructed vitamin D receptor-deficient (VDR-/-) and wild-type (WT) mouse models. *In vitro* experiments showed that 1,25(OH)2D significantly inhibited Th1 cell differentiation and the production of related cytokines (such as IL-2, IFN-γ, and TNF-β) activated by Bacillus Calmette-Guérin (BCG). *In vivo* experiments further demonstrated that in vitamin D-deficient mice vaccinated with BCG, 1,25(OH)2D reduced inflammatory infiltration in the spleen, decreased the expression of inflammatory cytokines, and promoted the development of Th2 cells. These results indicate that 1,25(OH)2D alleviates inflammatory responses by inhibiting Th1 cell differentiation and cytokine production through the JAK/STAT pathway (46), while fostering the expression of Th2 cell factors (IL-4, IL-5, IL-9, IL-13) (36, 43). Vitamin D also lowers the levels of IL-12 and IL-23, the Th1/Th17 polarizing cytokines produced by DCs, inhibits the differentiation of naive CD4^+^T cells into Th17 and Th1 cells, and significantly bolsters the development of FoxP3^+^CD127^low^CD25^+^ regulatory T cells (Tregs) and IL-10-producing T cells. The induction of ICOS^+^Tregs (mainly IL-10 producers), CD69^+^FoxP3^+^ and TIGIT^+^FoxP3^+^Tregs is also significantly increased (47). Moreover, 1,25(OH)2D curbs the expression of IL-17A, IL-22, TNF-α, IFN-γ and chemokine receptor CCR6 in Th17 cells, thereby stopping Th17 cells from migrating to inflamed tissues (48–50). It can also induce the differentiation of Tregs by promoting the expression of IL-10 and FoxP3, thereby curbing pro-inflammatory immune responses (51, 52).

VDR also exists in human B lymphocytes. Studies indicate that 1,25(OH)2D can curb the generation of plasma cells and memory B cells (53), downregulate CD40, NF-κB signaling, lessen the activation of human peripheral B cells and induce their apoptosis, and curb the production of IgE (54–56). Simultaneously, 1,25(OH)2D enhances the expression of IL-10 in activated B cells by recruiting VDR to the promoter of IL-10, thereby participating in the inhibition of T cell activation (57).

In summary, through various pathways to regulate the activity of various T cell subgroups and B cells, maintain immune balance and suppress inflammatory responses, vitamin D is of great significance for maintaining the stability of the immune system and preventing excessive immune responses.

## 3 Vitamin D and allergic diseases

### 3.1 Vitamin D and atopic dermatitis

Atopic dermatitis (AD) is a chronic, recurrent inflammatory skin allergic disease, characterized by the disorder of skin barrier function leading to dry skin, itching, eczematous skin lesions, and IgE-mediated sensitization to food and environmental allergens (58). In an ovalbumin (OVA)-induced AD mouse model (59), vitamin D significantly improved the skin condition of mice, decreased IgE and IL-5 levels, but increased IL-4 and IL-13 levels, reduced filaggrin levels, and decreased epidermal thickness. Histological studies further confirmed that vitamin D has significant effects in alleviating inflammation and improving the pathological state of the skin. Most studies support the negative correlation between vitamin D levels and AD. A case-control study by El Taieb et al. (60) found that the average vitamin D level in children with AD was much lower than the normal value. A nationwide cross-sectional survey conducted by Heimbeck et al. (61) in Germany found that low serum vitamin D levels were negatively correlated with eczema in German children and adolescents. Ahmed Mohamed et al. (62) also observed a dose-response relationship between vitamin D deficiency and the prevalence of AD in a comparison of 100 AD patients and 1001 normal controls in the dermatology outpatient department in Cairo, Egypt. Moreover, most studies have observed a negative correlation between the severity of AD and serum 25(OH)D levels; the more severe the vitamin D deficiency, the higher the scoring atopic dermatitis (SCORAD) score (63–67). A recent case-control study (64) involving 96 AD patients and 90 healthy controls found that compared with atopy and eosinophilia, the reduction of serum vitamin D levels seems to have a more significant impact on the severity of AD. For each unit increase in serum vitamin D levels, the SCORAD index decreases by 0.449 units, while an increase of 1 unit in eosinophil count will cause the SCORAD index to increase by 0.009 units. However, several other cohort studies believe that there is no association between the risk of AD in offspring at 3–5 years and the level of vitamin D during pregnancy, at birth, and early life (68–70). Overall, the majority of existing studies suggest that vitamin D is associated with the risk and severity of atopic dermatitis.

### 3.2 Vitamin D and allergic rhinitis

Allergic rhinitis (AR) is a common allergic disease mediated by immunoglobulin E (IgE), caused by inhaled allergens, and clinically manifested as sneezing, nasal congestion, nasal itching, and rhinorrhea. In an ovalbumin-induced AR mouse model, 1,25(OH)2D reduced serum levels of ovalbumin-specific IgE and spleen IL-17 levels, as well as IL-5 and IL-13 levels in nasal lavage fluid (71). Additionally, studies on human serum have shown that the level of 1,25(OH)2D is related to the Th1/Th2 balance in AR patients, and vitamin D deficiency shifts the Th1/Th2 balance to Th2 (72). Most studies believe that the serum vitamin D level of AR patients is lower than that of healthy people or the control group (73–78). Jung et al. (79) conducted a large-scale national survey of 8,012 Korean adults over 18 years old, indicating that the lower the 25(OH)D level, the higher the prevalence of AR. A recent secondary study (80) of the Vitamin D Antenatal Asthma Reduction Trial (VDAART) birth cohort showed that compared with patients with vitamin D deficiency in early and late pregnancy, the occurrence of AR and sensitization to airborne allergens at 3 and 6 years old in the offspring of mothers with sufficient prenatal vitamin D in late pregnancy was reduced (OR= 0.47; 95% CI, 0.26–0.84). Bunyavanich et al. (81) studied 1,248 mother-child pairs in the US prenatal cohort and found that every 100 IU/d of dietary vitamin D intake in the first three months and the last three months of pregnancy reduced the chance of school-age children suffering from AR by 21% and 20% respectively. Saad et al. (73) found in a cohort study of 120 Egyptian children with AR and 100 healthy children that the average 25(OH)D level of patients with moderate/severe AR was significantly lower than that of patients with mild AR, and the average 25(OH)D level of the AR group was negatively correlated with the total nasal symptom score and total IgE level. However, it has been observed that the association between Vitamin D and AR is affected by race, age, gender, etc. (82–84). For example, Mai et al. (84) reported that lower levels of vitamin D in the Norwegian adult population are related to an increased risk of AR in men and a reduced risk of AR in women. The authors speculated that this might be related to female sex hormones enhancing Th1 responses and reducing Th2 responses. Wegienka et al. (83) found that higher prenatal and cord blood 25(OH)D levels were generally associated with fewer allergic outcomes, such as eczema and sensitization to airborne allergens. This association was more significant in white children and less evident in black children. Additionally, they observed that 25(OH)D levels were negatively associated with sensitization to airborne allergens only in black children.

Some research has refuted the link between vitamin D and AR. A cross-sectional study conducted by Wu et al. (85), which included 32 patients with persistent AR and 25 controls, found no significant difference in serum 25(OH)D levels between the two groups. A large cross-sectional study (86) in Korea involving 15,212 adults aged 19 or above indicated, through multivariate linear regression analysis, that adults with vitamin D deficiency did not have an increased likelihood of asthma, AR, or IgE sensitization. A cohort study (87) collected the cord blood 25(OH)D levels of 239 newborns. Using a symptom questionnaire based on the International Study of Asthma and Allergies in Childhood (ISAAC) and following up these children until they were 5 years old, it was found that there was no correlation between cord serum 25(OH)D levels and asthma and AR at age 5. The most recent Mendelian randomization study (88) also did not find evidence of a causal relationship between serum vitamin D levels and AR risk in individuals of European descent. Therefore, more research is needed to confirm the relationship between vitamin D and the development of AR.

### 3.3 Vitamin D and asthma

Asthma is a common chronic respiratory disease, characterized by chronic inflammation of the airways and high airway reactivity, manifested as coughing, wheezing, chest tightness, and difficulty breathing. The most common phenotype is allergic asthma. Vasiliou et al. (89) investigated the immune responses and inflammatory markers in neonatal allergic airway disease using a vitamin D-deficient mouse model. Their findings indicated that vitamin D deficiency resulted in an elevated proportion of Th2 cells, a decrease in IL-10-secreting regulatory T cells, and exacerbated eosinophilic inflammation and airway remodeling following exposure to house dust mites, thereby fostering the development of allergic diseases (90). Vitamin D supplementation significantly mitigated these pathological changes. Hamzaoui et al. collected peripheral blood samples from young children with asthma and found that vitamin D significantly inhibited the differentiation of Th17 cells and the production of IL-17 while increasing the levels of the anti-inflammatory cytokine IL-10 (91). A cross-sectional study in the Cyprus region (92) included 69 active asthmatics and 671 never wheezing/never asthmatic teenagers aged 16–17. It was found that the average vitamin D level of asthmatic children was lower, and in the AA group, the vitamin D level was negatively correlated with the severity of asthma. Previously, Bener et al. (93) compared the vitamin D levels of 483 asthmatic children with healthy children in Qatar, and also proposed that vitamin D deficiency is a major predictor of childhood asthma. A cross-sectional study (94) in the UK of 435,040 adults found that compared with vitamin D deficiency, the risk of asthma in individuals with low and sufficient vitamin D concentrations was reduced by 6.4% and 9.8% respectively, and their lung function would also improve. Similarly, many studies have reported that 25(OH)D deficiency is related to increased risk of asthma in newborns, adolescents, adults, and decreased lung function (95–100), and is affected by many factors such as gender, race, ethnicity, smoking, whether to use ICS, sleep mode and genetic susceptibility (98–101). As Chang et al. (98) discovered in a large-scale prospective cohort study based on the UK Biobank, the protective effect of vitamin D against asthma was strongest under healthy sleep patterns. In individuals with moderate genetic risk, higher levels of vitamin D were associated with a significantly reduced risk of asthma. The protective effect of vitamin D was most notable in males, individuals under 60 years old, overweight individuals, and current or former smokers. Another Norwegian cohort study reported that the association between vitamin D levels and lung function varied by gender and allergy status, with this association being particularly significant among male asthma patients (99).

Studies have shown that vitamin D has a protective effect on airway smooth muscle cell contraction and remodeling in asthma. Vitamin D inhibits the growth of airway smooth muscle cells by reducing the expression of cyclin D1 and inducing the phosphorylation of retinoblastoma protein and checkpoint kinase 1 (102). It also inhibits vascular endothelial growth factor (VEGF)-induced ADAM Metallopeptidase Domain 33 (ADAM33) expression and proliferation, reducing airway remodeling (103). Furthermore, Plesa et al. demonstrated that vitamin D can inhibit the proliferation and migration of bronchial fibroblasts by suppressing ERK1/2 and Akt signaling pathways and upregulating genes involved in cell cycle arrest, such as p21 and p27. It also reduces the expression of genes involved in extracellular matrix remodeling, such as type I collagen and matrix metallopeptidase 2 (MMP2) (104). Vitamin D inhibits NF-κB activation, reducing the expression of pro-inflammatory cytokines like IL-6 and IL-8 (105), and decreases the expression of type I collagen and protein arginine methyltransferase 1 (PRMT1) activity, exhibiting anti-inflammatory and antifibrotic effects (106). These mechanisms indicate that vitamin D may play a pivotal role in regulating airway remodeling in asthma, thereby reinforcing its association with the condition and its potential as an adjunctive therapy for asthma management (107, 108).

Recent studies underscore a close interrelationship between vitamin D, gut microbiota, and asthma. Vitamin D deficiency may compromise barrier integrity and alter microbiome composition, with gut dysbiosis potentially impairing both local and pulmonary immune functions, thus heightening asthma susceptibility. Respiratory infections can disrupt the gut microbiome, decreasing bacteria that produce short-chain fatty acids (SCFAs), which in turn impacts the function and fate of immune cells, further exacerbating asthma symptoms (109–111).

Contradictorily, as mentioned earlier, *in vitro* experiments have proven that vitamin D can promote Th2 cell shift (45, 112), which seems to contradict the protective effect of vitamin D on allergies. A cohort study (113) based on a large population of adults only reported that vitamin D deficiency is related to acute asthma attacks, but there is no significant connection with doctor-diagnosed asthma. Cheng et al. (86) investigated the data of 15,212 people aged 19 and above in South Korea, and also found that adults with vitamin D deficiency did not increase the likelihood of asthma or IgE sensitization. Overall, the majority of studies support the association between vitamin D and the risk and severity of asthma.

### 3.4 Vitamin D and food allergies

Food allergies (FAs) are pathological reactions triggered by the immune system mistakenly identifying one or more protein antigens in food as harmful substances. Symptoms can accumulate in multiple systems such as the skin, digestion, respiration, circulation, and even lead to anaphylactic shock. They can be classified as IgE-mediated, IgE-dependent and IgE non-dependent pathways co-mediated (mixed), and non-IgE-mediated (114). Studies have shown that light, latitude, and season of birth are related to FAs (115). For example, a survey by Vassallo (116) and others showed that the proportion of children under 5 years old born in autumn or winter with FA is 50% higher than those born in spring or summer. In the United States and Australia, the overall risk of allergies, FAs, and FA markers in the population farthest from the equator is higher than those closest to the equator (117, 118). Seasons and latitude affect the exposure of the human body to sunlight and solar radiation (with fewer megajoules of sunlight per square meter in the world’s southernmost and northernmost parts and shorter daylight hours in autumn and winter). The synthesis of vitamin D is also related to light with 80%-90% of the serum 25(OH)D levels deriving from sun exposure. Its level changes periodically with the seasons, because the time for sun exposure to synthesize vitamin D is longer in winter than in summer (119). There is also direct evidence indicating that insufficient sunlight exposure before the age of 24 months may elevate the risk of developing FAs, asthma, AR, and AD in school-aged children (120). This connects vitamin D with FAs, AR, AD and other allergic diseases. A cross-sectional study (121) by Silva and others found that infants with cow’s milk protein allergy had lower average vitamin D levels compared with the healthy control group. A large study (122) reported a cross-sectional association between vitamin D deficiency (VDI; 25(OH)D <50 nmol/L) in one-year-old infants of Australian-born parents and positive provocation test IgE-mediated food allergies, with evidence suggesting a dose-response relationship, where infants deficient in vitamin D had a 3-fold increased risk of egg allergy, an 11-fold increased risk of peanut allergy, and a 10-fold increased risk in infants with two or more FAs. In addition, in infants who already have food sensitization, those who are deficient in vitamin D have a 6-fold risk of developing FAs compared to those who develop food tolerance. A recent systematic review suggests that maternal vitamin D deficiency and infant vitamin D deficiency appear to increase the risk of FAs, especially in the second year after the baby’s birth (123). In contrast, Weisse et al. (124) observed in a cohort study that the higher the maternal and cord blood 25(OH)D levels, the higher the risk of FAs in children in the first two years, and they believe that this association can be explained by the observed decrease in the number of Treg cells at birth. Similarly, a case-cohort study by Molloy (125) and others also believes that vitamin deficiency in the first 6 months of infancy is not significantly associated with FAs at one year old. In summary, most of the literature supports a significant association between vitamin D and food allergies, but the specific mechanism needs to be further studied.

Indeed, the relationship between vitamin D and allergies may depend on several factors, including an individual’s vitamin D levels, the type of allergic disease, gender, ethnicity, and other potential immune regulatory mechanisms. Therefore, further research is necessary to clarify the exact role of vitamin D in allergic diseases, and how to effectively use vitamin D in the clinic to regulate immune responses and improve the treatment of allergic diseases.

## 4 The clinical efficacy of vitamin D supplementation in allergic diseases

### 4.1 The impact of vitamin D supplementation on the outcomes of allergic diseases

With the established association between vitamin D deficiency and allergic diseases, numerous studies have been dedicated to investigating the clinical benefits of vitamin D supplementation in various populations, and the results have been relatively promising (**Table 1**). A significant, large-scale randomized controlled trial (RCT) study is the VDAART trial (126). The VDAART trial was a randomized, double-blind, placebo-controlled study conducted across three centers in the United States. It included 881 non-smoking pregnant women aged between 18–39 years, who were at 10–18 weeks of gestation and had a high risk of their offspring developing asthma. These women were randomly divided to receive either the intervention group (4400 IU of vitamin D daily) or placebo (a multivitamin containing 400 IU of vitamin D daily) until childbirth. The study examined the maternal 25(OH)D levels in the late stages of pregnancy and the conditions of asthma and recurrent wheezing in the offspring. While the intention-to-treat analysis and stratified analysis based on the 25(OH)D levels of the mothers during pregnancy indicated that maternal vitamin D supplementation did not impact the occurrence of asthma and recurrent wheezing in the offspring at ages 3 and 6 (127, 128), further analysis of early and late prenatal vitamin D status, baseline vitamin D levels of the mothers at the beginning of the study, and the timing of supplementation initiation led researchers to conclude that adequate prenatal vitamin D throughout pregnancy provides a protective effect against the development of asthma/recurrent wheezing in children before the age of 3 (129). The study also found that earlier intervention during pregnancy can significantly reduce the risk of asthma or recurrent wheezing in offspring, with each week of earlier intervention reducing the odds of the offspring developing asthma and recurrent wheezing by 15%. When compared with daily supplementation of 400 IU of vitamin D, initiating daily intake of 4400 IU of vitamin D between the 9th and 12th weeks can decrease the odds of asthma or recurrent wheezing by a maximum of 55% (130). Concurrently, a secondary analysis of VDAART by Chen et al. (80) also highlighted that prenatal vitamin D supplementation has a protective effect on the incidence of AR and sensitization to airborne allergens at ages 3 and 6.

**Table 1:** Studies on the impact of vitamin D supplementation on allergy outcomes.

Reference	Design	Country	Sample size and age	Subject characteristics	Treatment	Primary outcome	Conclusion
Litonjua et al. (2014,2016,2020) (126–128)	Multicenter, double blind, randomized, placebo-controlled trial	Boston, Massachusetts; St Louis, Missouri; San Diego, USA	n=806, vitamin D group: 27.5(5.5) y; Placebo group: 27.32(5.5) y	Pregnant women with either history of asthma or allergies in themselves or the biological father	Vitamin D group: vitamin D3 4400 IU/day; Placebo group: vitamin D3 400 IU/day, duration the woman’s pregnancy, about 22 to 30 weeks	Offspring asthma or recurrent wheeze	• Prenatal VD supplementation alone • Has no effects on offspring asthma and recurrent wheeze development up to age 6
Lu et al. (2021) (129); Shadid et al. (2023) (130)	Multicenter, double blind, randomized, placebo-controlled trial, secondary analysis	Boston, Massachusetts; St Louis, Missouri; San Diego, USA	n=806, vitamin D group: 27.5(5.5) y; Placebo group: 27.32(5.5) y	Pregnant women with either history of asthma or allergies in themselves or the biological father	Vitamin D group: vitamin D3 4400 IU/day; Placebo group: vitamin D3 400 IU/day, duration the woman’s pregnancy, about 22 to 30 weeks	Offspring asthma or recurrent wheeze	•VD sufficiency throughout pregnancy • Reducing the risk of asthma and recurrent wheeze in offspring •The earlier the intervention, the better the effect
Chen et al. (2021) (80)	Multicenter, double blind, randomized, placebo-controlled trial, secondary analysis	Boston, Massachusetts; St Louis, Missouri; San Diego, USA	n=806, vitamin D group: 27.5(5.5) y; Placebo group: 27.32(5.5) y	Pregnant women with either history of asthma or allergies in themselves or the biological father	Vitamin D group: vitamin D3 4400 IU/day; Placebo group: vitamin D3 400 IU/day, duration the woman’s pregnancy, about 22 to 30 weeks	Offspring aeroallergen sensitization and allergic Rhinitis	•VD sufficiency throughout pregnancy •Attenuating the risk of offspring allergic rhinitis with sensitization by age 6 years.
Andújar-Espinosa et al. (2021) (131)	Prospective, randomized, triple-blind, placebo-controlled, parallel-group study	Murcia, Spain	n=112, Calcifediol group: 54.57(15.83) y; Placebo group: 56.61(15.00) y	Adult asthmatic patients with serum 25(OH)D3 <30 ng/mL	25(OH)D group: 25(OH)D 16000 IU/week; Placebo group: placebo + usual asthma treatment, 6months	Asthma control degree: ACT; Life quality: Mini-AQLQ, Asthma attacks, Oral corticosteroid cycles, Emergency visits, Unscheduled consultations with the primary care physician and hospitalizations for asthma.	•Weekly oral calcifediol compared with placebo •Improving asthma control among asthmaic adults with VD deficiency at 6 months.
Nabavizadeh et al. (2023) (132)	Prospective, randomized, double-blinded clinical trial	Shiraz, Iran	n=69, vitamin D group: 27.5(5.5) y; Placebo group: 27.32(5.5) y	Patients with chronic spontaneous urticaria	Low vitamin D3 group: 4200 IU/week; High vitamin D3 group: 28,000 IU/week, 12 weeks	Quality of life (CU-Q2oL questionnaire), urticaria severity (USS questionnaire) and medication scores	•High dose of vitamin D • Reducing CU symptoms severity and the required doses of allergy medications.
Mohamed et al. (2022) (133)	Prospective, randomized, controlled and single blinded clinical trial	Cairo, Egypt	n=77, Study group: 35.2(4.37) y; Placebo group: 34.6(9.8) y	Adults >18 y with urticaria episodes at least 2 days per week for 6 weeks or longer	Study group: 0.25μg alfacalcidol + Hydroxyzine 25 mg/day; Placebo group: 0.25μg placebo + Hydroxyzine 25 mg/day, 12 weeks	UAS7 total score, serum IL-6, hsCRP, TNF-α	•VD supplementation for 12 weeks •Improveing UAS7 total score and the level of the inflammatory markers •Having a beneficial effect on CSU patients
El-Heis et al. (2022) (134)	Multicenter, double-blind, randomized placebo-controlled trial	Southampton, Oxford and Sheffield, UK	n=703, Cholecalciferol group: 31.0(4.9) y; Placebo group: 31.1(5.0) y	Pregnant women aged over 18 years, gestational age < 17 week, and serum 25(OH)D between 25 and 100 nmol/L and calcium < 2.75 mmol/L	Cholecalciferol group: cholecalciferol 1000 IU daily; Placebo group: matched placebo; from 14 weeks’ gestation until delivery	Offspring atopic eczema at ages 12, 24 and 48 months	•Maternal cholecalciferol supplementation •Reducing the risk of atopic eczema in offspring during their first year of life.
Aldaghi et al. (2022) (135)	Single-center, double-blind, randomized, parallel-group clinical trial	Sabzevar, Iran	n=81, Synbiotic group: 4.09(2.78) y; vitamin D3 group: 4.44(2.84) y; Control group: 6.07(4.50) y	Infants under 12 months of age, without other chronic diseases, SCORAD score>14	Synbiotic group: synbiotic 5 drops/day +routine treatment; vitamin D3 group: vitamin D3 1000IU/day + routine treatment; Control group: routine treatments, 2months	SCORAD score	•VD supplements administration along with routine treatments •Reducing the severity of AD in infants.
Mansour et al. (2020) (136)	Double-blind, randomized, parallel, placebo-controlled clinical trial	Cairo, Egypt	n=86, vitamin D group: 12(4.75) y; Placebo group: 11(5.5) y	Patients aged from 5 to 16 years old, with a diagnosis of severe AD	Vitamin D3 group: vitamin D3 1600 IU/day + 1% hydrocortisone cream twice daily; Placebo group: placebo + 1% hydrocortisone cream twice daily, 12 weeks	EASI score	•VD supplements in children with severe AD •Providing clinical improvement
Cabalín et al. (2023) (137)	Open-label pilot trial	Santiago, Chile​	n=86, 6.8(3.8) y	Children aged 2–18 years with AD, SCORAD ≥ 25	Oral doses of liquid VD3 8000 IU/week for 2–5.9 years; 12,000 IU/week for 6–11.9 years; 16,000 IU/week for 12–18 years, 6weeks	Stratum corneum RNA expression of the VDR, CAMP, and TSLP genes, and LL-37 protein	•VD supplementation in children with AD. •Improving AD severity, VDR and Cathelicidin expression in lesional skin
Guo(2023) (138)	Single-center, Assessor/statistician-blinded, randomized, parallel study	Ganzhou, China	n=128, Experimental group: 32.8(10.2) y; Control group: 32.1(11.1) y	Patients aged between 16 and 60 years, with moderate-to-severe AR, did not receive any AR-related treatment within two weeks of diagnosis, had good drug compliance	Experimental group: vitamin D 1600 IU/day + 200 μg mometasone nasal spray twice/day; Control group: 200 μg mometasone nasal spray twice/day, 4weeks	TNSS, RQLQ, T lymphocyte subsets (CD3+, CD4+ and CD8+), IL-10, TNF-α, and IFN-γ	•VD supplementation in AR patients •Improving AR symptoms and quality-of-life •Decreasing TNF-α levels and increased IFN-γ and IL-10 levels.
Liu et al. (2022) (139)	Single-center, randomized, controlled trial	Hohhot, China	n= 90, vitamin D group: 27.2(8.8) y; DCD group: 27.3(7.1) y; Control group: 31.2(10.6) y	Patients with mild seasonal pollen AR	Vitamin D group: oral desloratadine citrate disodium (DCD, 8.8 mg/day) + vitamin D3 nasal drops (1.5 × 106 IU, once/week; DCD group: DCD, 8.8 mg/day; Control group: no medication	Peripheral blood eosinophils, IL-4 levels, and nasal symptoms	•VD3 as an adjuvant therapy •Alleviating the nasal symptoms and decrease serum IL-4 and blood eosinophil count in patients with AR.

VD, vitamin D; ACT, asthma control test; mini-AQLQ, the mini asthma quality of life questionnaire; CU-Q2oL, chronic urticaria quality of life questionnaire; USS, urticaria severity score; CU, chronic urticaria; CSU, Chronic spontaneous urticaria; UAS7, urticaria activity score over 7 days; AD, atopic dermatitis; SCORAD, scoring atopic dermatitis; EASI, eczema area and severity index; VDR, vitamin D receptor; CAMP, cathelicidin antimicrobial peptide; TSLP, thymic stromal lymphopoietin; AR, allergic rhinitis; TNSS, total nasal symptom score; RQLQ, rhinoconjunctivitis quality of life questionnaire.

A randomized, triple-blind, parallel, placebo-controlled study (131) conducted in Spain included 112 patients with an average age of 55 years suffering from asthma and with serum 25(OH)D levels below 30ng/mL. The study period was 6 months. The intervention group received 16,000 IU of oral cholecalciferol supplements weekly, while the control group added a placebo to the routine asthma treatment. The results showed that compared with the placebo, weekly oral supplementation of 25(OH)D can significantly improve Asthma Control Test (ACT) scores within 6 months. It can also improve the quality of life of patients, reduce the use of oral corticosteroids and the number of asthma attacks, and reduce the risk of hospital treatment for asthma.

In a prospective double-blind study conducted by Nabavizadeh et al. (132), 80 patients with chronic spontaneous urticaria were included. These patients were given low-dose vitamin D (4200IU/week, Group 1) and high-dose vitamin D (28,000 IU/week, Group 2) supplements for 12 weeks, in addition to their baseline treatment plan. The results indicated that both groups experienced a significant decrease in the total scores of urticaria severity, medication scores, and quality of life scores. Moreover, the high vitamin D group exhibited a more significant reduction in the total score of urticaria severity at the 6th week, and a more noticeable decrease in the quality-of-life score at the 6th and 12th weeks, compared to the low vitamin D group. Another study by Mohamed et al. (133), which focused on adults aged 18 and above in Egypt, corroborated these findings. They also observed that, in comparison with the placebo group and baseline results, the study group had significantly lower average serum IL-6, hypersensitive C-reactive protein (hs-CRP), and TNF-α levels.

For AD, El-Heis et al. (134) observed that supplementing mothers with 1000 IU of vitamin D daily from 14 weeks of pregnancy to delivery could reduce the incidence of AD in the first year after birth. Most RCT studies have confirmed that the addition of vitamin D to the basic treatment of AD significantly reduces the severity of the disease in children, including reducing SCORAD scores and eczema area and severity index (EASI) scores (135, 136). A study in the United States further found that oral vitamin D supplementation may be related to the increase in the expression of VDR and Cathelicidin in lesion skin (137).

In the context of AR, an RCT study carried out by Guo et al. (138) discovered that supplementing vitamin D can enhance the therapeutic effect of mometasone nasal spray on moderate to severe AR. This resulted in a more significant decrease in patients’ TNSS total score, T lymphocyte subsets (CD3+, CD4+), CD4+/CD8+ ratio, TNF-α, and rhinoconjunctivitis quality of life questionnaire (RQLQ) total score. The levels of CD8+, IFN-γ, IL-10, and serum vitamin D were found to be more significantly increased compared to the control group and the initial test. Liu et al. (139) also noticed that patients who received vitamin D as an adjunct therapy had higher serum 25(OH)D levels, lower AR symptom scores, IL-4 levels, and peripheral blood eosinophils, and a higher effective rate of AR treatment, compared to those treated with desloratadine citrate dihydrate (DCD) alone. Hence, supplementing vitamin D in routine treatment can serve as an effective adjuvant treatment for AR patients by suppressing inflammation (**Table 1**).

The above studies have many limitations. First, the study population may be single-center, short-term, and small-scale. Second, the selection of the severity of the study subjects may be overly broad. Third, most studies may not consider the intake of vitamin D in the diet and the data of the patient’s sun exposure time. Fourth, many self-filled questionnaires may have recall bias. Therefore, subsequent studies should consider the impact of differences in age, gender, severity, race, etc. on the clinical efficacy of vitamin D supplementation, and further large-scale, long-term follow-up, multi-center clinical trials and randomized controlled trials are needed. Also, it’s necessary to determine the optimal dosage and duration of vitamin D supplementation and to deeply understand the impact of vitamin D on the treatment effect of allergic diseases.

### 4.2 Vitamin D and allergen immunotherapy

Allergen immunotherapy (AIT) is a therapeutic approach for allergic diseases that modulates the patient’s immune system by progressively increasing the dose of allergens, thus reducing the allergic response to specific allergens. This method is commonly used for treating conditions such as pollen allergy, house dust mite (HDM) allergy, certain FAs, and bee venom allergy. AIT can be administered via subcutaneous injections, sublingual drops, or sublingual tablets. It can decrease allergen-specific Th2, stimulate regulatory T cells and B cells, and produce IgG and IgA blocking antibodies, thereby inducing tolerance to allergens in patients, reducing symptoms, and enhancing the quality of life. Given its long treatment cycle and high demand for patient compliance, new strategies are being explored currently, such as novel adjuvants, recombinant allergens, and immunomodulators, to provide safer, more effective, and convenient treatment plans and more lasting long-term tolerance (140). In this context, vitamin D has been identified as a potential enhancer, improving the effectiveness of AIT.

Numerous animal studies have demonstrated the enhancing effect of vitamin D on AIT. In a murine model of grass pollen-induced allergic asthma, vitamin D supplementation reduced the Th2 cell factor responses and innate cell factor responses to allergens in lung tissue, increased IL-10 in lung tissue, and reduced airway hyperresponsiveness (AHR). Researchers observed that, compared to subcutaneous immunotherapy (SCIT) or sublingual immunotherapy (SLIT) alone, the combination of 1,25(OH)2D with SCIT or SLIT resulted in a more significant reduction in eosinophil counts and IL-5 and IL-13 levels in bronchoalveolar lavage fluid, as well as marked improvement in lung function. The authors concluded that vitamin D enhances the efficacy of grass pollen SLIT and SCIT in mice (141, 142).

Li (143) and colleagues conducted a regression analysis on 153 AR patients who received SLIT, revealing that a deficiency in serum Vitamin D could impact the effectiveness of SLIT in children with AR. Majak (144) and others carried out a retrospective secondary analysis of the combined data from a prospective, randomized, placebo-controlled trial involving 36 children with asthma undergoing AIT. They discovered that patients with higher serum 25(OH)D levels experienced more significant reductions in asthma symptom scores and AIT-induced corticosteroid reduction effects over the 12-month AIT period. These patients also exhibited higher peripheral blood TGF-β production and greater expression of Foxp3 positive cells, suggesting that vitamin D might serve as an effective adjuvant for AIT. A randomized, double-blind, placebo-controlled trial (145) in Poland, which included 50 children aged 5–12 who were allergic to grass pollen and had AR (with 8 also having asthma), used a daily 5-grass pollen sublingual 300 IR tablet and supplemented with either 1000 IU of vitamin D or a placebo for 5 months. The study found that the SLIT plus vitamin D group was more effective in alleviating nasal symptoms, asthma symptoms, and symptom-medication combined scores compared to the placebo group. In a study on children with asthma who were allergic to HDMs, the SCIT plus vitamin D group had a lower total asthma symptom score at the 6th month and the highest average fluorescence intensity of Foxp3 at the 12th month, compared to using SCIT alone (146).

A study conducted in Bangkok, Thailand (147), demonstrated that, compared to a placebo, adult patients allergic to HDMs who received subcutaneous AIT and supplemented with vitamin D experienced significantly reduced symptom-drug scores and increased treatment response rates. This improvement in allergic symptoms is thought to be achieved by vitamin D significantly reducing the quantity of dysfunctional regulatory T cells (CRTH2+Treg). This lends further support to the potential value of vitamin D in AIT. These findings also offer new treatment strategies for AIT and pave the way for new possibilities in the treatment of allergic diseases.

## 5 Summary

Based on existing research, the role of vitamin D in allergic diseases cannot be ignored. Vitamin D can affect the occurrence and development of allergic diseases through its immune regulatory function. Although existing research shows that vitamin D deficiency is related to an increased risk of allergic diseases, its correlation is not consistent among different populations, and the effect of vitamin D supplementation on improving the outcomes of these diseases still needs further research. In terms of mechanism, there are many contradictions in vitamin D’s regulation of Th1/Th2 balance, Th17/Treg, ILC2 cells, etc. Due to the complexity of the immune system, the occurrence and development of allergies by vitamin D cannot be explained by a single regulatory method, and further research is needed to discuss which regulation predominates. In terms of clinical efficacy, future research should explore the optimal supplement dose and duration of vitamin D more deeply, considering patients’ lifestyles, dietary habits, and basic health conditions, and carry out more rigorous and detailed research design. This includes cross-racial and regional studies, as well as analyses of different age and gender groups, to ensure the wide applicability and accuracy of the research results, and how to use vitamin D more effectively to regulate immune responses and improve the treatment effects of patients with allergic diseases. At the same time, we need to pay attention to the potential of vitamin D as an adjuvant combined with AIT, to develop safer, more effective and convenient treatment methods. In summary, vitamin D plays an important role in the prevention and treatment of allergic diseases, but its specific mechanisms and application strategies still need to be clarified by further research in the future.

## Author contributions

PZ: Writing – original draft, Writing – review & editing. QX: Writing – review & editing. RZ: Conceptualization, Supervision, Writing – review & editing.

## Glossary

**Table:**

AD	Atopic Dermatitis
AR	Allergic Rhinitis
AA	Allergic Asthma
VDR	Vitamin D Receptor
DCs	Dendritic Cells
ILCs	Innate Lymphoid Cells
GM-CSF	Granulocyte-Macrophage Colony-Stimulating Factor
CXCR4	C-X-C Motif Chemokine Receptor 4
MBP	Major Basic Protein
EPX	Eosinophil Peroxidase
ECP	Eosinophil Cationic Protein
EDN	Eosinophil-Derived Neurotoxin
NETs	Neutrophil Extracellular Traps
AECOPD	Acute Exacerbation of Chronic Obstructive Pulmonary Disease
ADCC	Antibody-Dependent Cellular Cytotoxicity
RXR	Retinoid X Receptor
CRIg	Complement Receptor Immunoglobulin
LPS	Lipopolysaccharide
INF-α	Interferon-Alpha
TGF-β	Transforming Growth Factor Beta
MHC	Major Histocompatibility Complex
TNF-α	Tumor Necrosis Factor Alpha
IFN-γ	Interferon-Gamma
IL	Interleukin
CCL17	C-C Chemokine Ligand 17
FOXP3	Forkhead Box P3
Th1	T Helper Cell 1
Th2	T Helper Cell 2
Th17	T Helper Cell 17
Tregs	Regulatory T Cells
SCORAD	Scoring Atopic Dermatitis
JAK/STAT	Janus Kinase/Signal Transducer and Activator of Transcription
ICOS	Inducible T-Cell Costimulator
CCR6	C-C Chemokine Receptor Type 6
NF-κB	Nuclear Factor Kappa-Light-Chain-Enhancer of Activated B Cells
IgE	Immunoglobulin E
SCFAs	Short-Chain Fatty Acids
AHR	Airway Hyperresponsiveness
ERK1/2	Extracellular Signal-Regulated Kinase 1/2
PRMT1	Protein Arginine Methyltransferase 1
VEGF	vascular endothelial growth factor
MMP2	matrix metallopeptidase 2
ACT	Asthma Control Test
hs-CRP	High-Sensitivity C-Reactive Protein
EASI	Eczema Area and Severity Index
RQLQ	Rhinoconjunctivitis Quality of Life Questionnaire
DCD	Desloratadine Citrate Dihydrate
AIT	Allergen Immunotherapy
SCIT	Subcutaneous Immunotherapy
SLIT	Sublingual Immunotherapy
HDM	House Dust Mite
